# Sleeping in a bubble: factors affecting sleep during New Zealand’s COVID-19 lockdown

**DOI:** 10.1093/sleepadvances/zpac017

**Published:** 2022-05-16

**Authors:** Rosemary Gibson, Harshi Shetty, Mikaela Carter, Mirjam Münch

**Affiliations:** School of Psychology, Massey University, Palmerston North, New Zealand; Sleep/Wake Research Centre, Massey University, Wellington, New Zealand; Sleep/Wake Research Centre, Massey University, Wellington, New Zealand; Sleep/Wake Research Centre, Massey University, Wellington, New Zealand; Sleep/Wake Research Centre, Massey University, Wellington, New Zealand; Research Centre for Hauora and Health, Massey University, Wellington, New Zealand; Centre for Chronobiology, University Psychiatric Clinics, Basel, Transfaculty Research Platform Molecular and Cognitive Neurosciences, Switzerland

**Keywords:** anxiety, circadian rhythms, dreams, sleep deprivation

## Abstract

New Zealand (NZ) enforced a rigorous lockdown in response to the outbreak of COVID-19 in 2020. Infection rates remained remarkably low, yet social and personal routines were affected. Factors associated with reporting worsening sleep were explored using an anonymous online survey launched during New Zealand’s 2020 lockdown. Participants were 723 adults aged 20–85 years (median: 45 years, 82% women). Bed and wake times occurred significantly later compared to pre-lockdown estimates and resulted in shorter social jetlag (15 min). During lockdown, 54.5% were identified as “poor sleepers” [i.e. score > 5 on the Pittsburgh Sleep Quality Index (PSQI)]. Overall, 45% rated their sleep quality to worsen compared to pre-lockdown, 22% reported an improvement. Reports of worsening sleep were significantly related to increased sleep latency, reduced sleep efficiency, and heightened PSQI scores compared to those with better sleep or no change. Subjectively worse sleep was significantly associated with less time engaging in physical activity, less exposure to daylight, and social interactions compared to pre-lockdown estimates (*p* < .05). Logistic regression models identified significant relationships between having more vivid dreams and worsening sleep. Worse sleepers also had increased likelihoods of reporting poorer mood and they also scored higher for anxiety compared to those with no change or improved sleep during lockdown (*p* < .05). Pandemic-related restrictions contributed to poorer self-reported sleep which was linked to deterioration of mood. Negative affect was comparatively lower than reported elsewhere. These findings provide unique insights to the psychosocial impact of the initial COVID-19 lockdown in New Zealand, where the disease outbreak remained low.

**Statement of Significance**

Sleep disturbances have increasingly been recognised as a problem with COVID-19 pandemic restrictions. However, this is the first study to present sleep-related changes among those living in New Zealand who endured an early and strict lockdown in 2020, restraining the disease outbreak to one of the lowest numbers worldwide. Therefore, the present findings are unique in that they relate to the impact of the pandemic-related social restrictions during a time and place of low infection rates. This work could inform future work about the importance of addressing and supporting sleep and mental health in general crisis situations.

## Introduction

Sleep health is increasingly recognised as multifaceted. While individual biology and behaviours are key, sleep is also driven at the social level. For example, the schedules of work or school, social engagements, and the regular exposures to time cues, which are further informed by the order and dynamics of wider society [1]. Therefore, when the patterns of daily life are interrupted by crisis at the micro or macro level, sleep is also affected.

During the COVID-19 pandemic, most countries moved into states of emergency with social distancing and shelter-in-place orders enforced. Daily life, schedules, and society as we knew it underwent rapid unsolicited change. Such changes challenged the social drivers of sleep and overall wellbeing [2]. Several studies have taken place examining the impact of the pandemic on sleep (for recent reviews see [3, 4]). Analysis of data from wearable devices as well as survey studies shows marked changes of sleep timing and sleep regularity during COVID-19 lockdowns. Most specifically, a tendency for sleep timing to shift later, for increased time spent in bed, and reduced variability in sleep timing between workdays and free days [5–8]. Such shifts have been associated with less social restrictions on sleep across the week including reduced or flexible work hours, reduced commutes, and increased opportunities for time spent outside and physical activity to support sleep preferences.

Despite trends of greater opportunity to sleep within lockdowns, a deterioration of sleep quality and symptoms of insomnia have also been reported. Such symptoms have been associated with the negative impact that the pandemic has had on mental wellbeing [9–11]. Increased anxiety, depression, fear, and loneliness have all been reported in relation to disease risk as well as social restrictions and living status [2, 11] which also have bidirectional relationships with sleep [10, 12]. Increased COVID-related stress has been linked with having longer sleep latency, more fragmented sleep, and nightmares compared to those with unchanged or lower stress [10, 13]. Findings which are akin to of the effects of natural disaster and trauma [14].

In March 2020, Aotearoa/New Zealand (NZ) enforced one of the world’s most rapid and strict lockdowns relative to disease outbreak [15]. This resulted in a stunted transmission of COVID-19 and lower risk of contracting the virus compared to other countries that had less ridged measures. Between NZ’s first case (February 28, 2020) and it’s returning to a state of reduced state of lockdown (Level One, June 8, 2020), 1500 cases were diagnosed and 22 COVID-related deaths recorded in NZ’s population of 5 Million [16]. This study used an anonymous online survey to investigate subjective sleep status, wellbeing, and mood during the initial NZ COVID-19 lockdown, as well as retrospectively (pre-lockdown). This enabled an exploration into the impacts of rigorous social restrictions triggered by a governmental response to the pandemic on subjective sleep status, wellbeing, and mood in a population who, at the time, also had reduced exposure or threat of illness. In particular, this study sought to:

1. Compare pre-lockdown estimates of subjectively assessed sleep timing with current (during lockdown) estimates;
2. Describe self-reported status and changes in sleep quality with lockdown;
3. Determine factors associated with self-reported worse or better sleep including environmental factors and behaviours (daylight exposure, physical activity) as well as mood, loneliness, dreaming, chronotype, and social jetlag

## Methods

On March 25, 2020, NZ moved into full (Level 4) lockdown with all but essential services remaining open, and the population being required to remain within their household “bubble” for 33 days. Reduced restrictions allowed at level 3 that direct social contacts could be expanded to one other family, whereas schools and workplaces remained closed to all but essential workers, and gatherings were restricted to 10 people. Reduced restriction at Level 2 permitted public gatherings to up to 100 people and schools and workplaces reopened with social with distancing measures in place. The country returned to a level of ‘normality’ in Level 1 (open except international borders) on June 8, 2020 [15].

An online survey was designed and launched via Qualtrics on April 11, 2020 to collect cross-sectional data on the sleep and wellbeing of adults living in NZ during four weeks of the most severe lockdown restrictions (National Alert Levels 4 and 3) [17] as well as retrospective, pre-lockdown estimates. The survey was advertised via a press release and subsequent media engagement (including national television and radio). A link was shared across email networks and promoted using free services on social media platforms (Twitter and Facebook).

### Measures

The 57-item survey included demographic details (e.g. age, sex, region, ethnicity, living, working, and family situations) and health status, including self-rated quality of health, diagnosis of a physical or mental health condition, and alcohol, caffeine, and tobacco consumption. Current work pattern was categorised as working shifts or variable hours (including those reporting working shifts with or without nights or irregular/variable work patterns) and daytime work without shifts. Current and pre-lockdown sleep timing on free and workdays, was gathered using the ultra-short version of the Munich Chronotype Questionnaire (μMCTQ) [18]. Mid-sleep times were derived as the midpoint (i.e. clock time) between sleep onset (i.e. bedtime minus self-estimated sleep latency) and wake time (separately for free and workdays). Mid-sleep time is widely used as a proxy for circadian phase and to assess chronotype. Estimates of time spent in waking activities (daylight exposure, physical activity, social engagement, and checking news) were also obtained concerning current and pre-lockdown situation.

A subjective change of sleep quality during lockdown was considered key. This was assessed by using the general question “How has your general sleep quality changed since the beginning of the COVID-19 pandemic restrictions?” with three options: “better “, “worse”, or “no change”. Current sleep quality (i.e. during lockdown) was further described using the Pittsburgh Sleep Quality Index (PSQI) [19]. Additional items were included from the Mannheim dream questionnaire [20] to measure dream frequency and intensity. Mood items included the Hospital Anxiety and Depression Scale (HADS) [21], self-rated changes in mood (‘How has your mood changed since the beginning of the lockdown?’ with three options: “better”, “worse”, or “no change”), indicators of social and emotional loneliness (Gierveld’s loneliness scale) [22], as well as self-rating loneliness in the past week (from the General Social Survey) [23].

Demographic and sleep-related items (including PSQI) as well as the μMCTQ were mandatory, while more sensitive questions concerning mood, anxiety, and loneliness were optional. Open-ended questions concerning sleep and mood were also included to provide opportunities for participants to elaborate on their experience if they wanted to (to be presented elsewhere). The survey took a median of 24.9 min to complete (IQR = 16.6 min). Informed consent was implied by completion of the survey. All data were anonymous except in cases where participants chose to share their contact details for inclusion on a mailing list for a summary of results or future research opportunities. Online information stated that participation in the survey resulted in implicit consent. Ethical approval was obtained from the Massey University Northern Ethics Committee (NOR 20/14).

### Data analyses

Only surveys with complete and valid data pertaining to self-reported change in sleep status as well as μMCTQ, PSQI were included. According to this criteria, 723 data sets were included (65% of the 1120 initial responses). All standard questionnaires were scored according to recommended guidelines.

The primary (discrete) outcome variable was subjective change in sleep quality since the beginning of the COVID-19 pandemic restrictions. (The number of participants in each of the three groups (“better”, “worse”, or “no change”) were used for the univariate analyses (Kruskal–Wallis or Chi square tests) to identify factors asssociated with sleep quality changes during lockdown.

Sleep timings and durations (µMCTQ) were averaged for workdays and free days pre- and during lockdown. Mid-sleep time was also corrected for sleep duration on free days (MSFsc), and ‘social jetlag’ was calculated as difference between mid-sleep timing on free days and workdays [18]. Absolute and weighted totals (i.e., taking into account the number of work- and free days) of sleep timing data were calculated and compared using Wilcoxon signed ranks tests. Time spent with waking activities (i.e. daylight exposure, physical activity, social interaction, media engagement) was also averaged and compared pre- and during lockdown. Lockdown sleep status was further described using the PSQI global sleep quality score (0: no sleep problem to 21: severe sleep problem) with “poor sleepers” defined as those scoring >5 [19]. Dreaming recall was scored on the 7-item scale: from 0 (never) to 6 (every morning), and dream intensity on a 5-items scale between 0: not at all and 4: very intense [20].

The items from the HADS were summed to provide global scores for both anxiety and depression (i.e. a score between 0 and 21) with those scoring ≥8 categorised as “borderline - heightened risk” [21]. Loneliness was defined using a single item from the General Social Survey [23] concerning how much of the past week participants had felt lonely (from 0: none to almost none of the time, to 4: all or almost all of the time) as well using a global social and emotional loneliness score from the Gierveld’s loneliness scale (from 0: not lonely, to 6: severe loneliness) [22]. The self-reported worse mood since beginning of lockdown was also used to identify significant relationships with sleep changes. Due to small numbers of participants in some categories, not all variables could be considered (e.g. ethnicity or essential worker status). All variables with significant univariate associations (*p* < .05) were considered in further multivariate models.

Binomial regression models were used to explore the factors independently associated with reporting worse sleep quality during lockdown (as opposed to having no change or better sleep). Final models were decided upon after consideration of several iterations. Firstly, including demographic and pre-existing health variables only; secondly, adding in waking activities; thirdly, adding in sleep timing, quality, and dreaming; and fourthly, adding in mood and loneliness items. Final models included 72.8% of participants due to missing data for some variables. Data pertaining to changed timing in waking activities and sleep were log-transformed prior to analysis if they were not normally distributed. Variables were used in their continuous rather than categorical form where available (e.g. the PSQI and HADS scores) to enhance model specificity. All analyses were undertaken in SPSS software version 26.0 (SPSS Inc. Chicago, IL, USA).

## Results

### Participant characteristics

Participants were 723 adults living across NZ during the initial 2020 lockdown. Most were living in the more densely populated regions of Auckland (26.0%) and Wellington (39.7%) and lived in stand-alone houses (80.9%) with access to a garden or balcony (96.5%). The age range was broad (20–85 years, median 45 years), the majority (82.3%) were female, and 64.8% identified as being of NZ European ethnicity (6.8% identified as Māori). Almost half of participants lived alone (45.9%), but the number of co-residents ranged from 1 to 12, (with 5% living with more than 3 others). Most participants were highly educated (76.3% had tertiary education), 57.4% were in full time work, and the majority (80.8%) worked a stable daytime non-shift working pattern. “Essential workers” represented 11.6% of the sample. Most participants rated themselves as in very good to excellent health (68.6%). However, 44.5% had either a chronic health condition or mental illness (see Table 1).

**Table 1.** Distribution of key demographic, health status, waking activities, sleep status, and mood of the total sample and split by self-rated change in sleep during lockdown compared to pre-lockdown (Kruskal–Wallis and Chi square tests indicating changes between better sleep, no change, and worse sleep)

	Total				Better sleep (158, 21.9%)				No change (240, 33.2%)				Worse sleep (325, 45.0%)				Significance
Variable	N	%	Mdn	IQR	N	%	Mdn	IQR	N	%	Mdn	IQR	N	%	Mdn	IQR	*P*
Age (years)	721		45.00	22.00	158		48.00	23.00	238		46.00	25.00	325		41.00	18.00	*** ^a,b^
Female	723	82.3%			158	84.0%				76.6%				87.3%			* ^d^
Works shifts or variable hours	617	16.7%			146	16.4%			202	22.8%			269	12.3%			* ^e^
Health Status (excellent/very good)^†^	723	68.6%			158	75.3%			240	76.7%			325	59.4%			*** ^d^
Chronic disease diagnosed	723	27.7%			158	29.7%			240	27.9%			325	27.1%			NS
Mental Illness diagnosed	723	33.2%			158	31.0%			240	21.7%			325	42.8%			*** ^d^
**Changed activities & sleep during lockdown vs. pre-lockdown:**																	
Daylight exposure (h)	723		0.00	1.50	158		0.00	1.50	240		0.00	1.50	325		-0.50	1.53	*** ^a,c^
Physical activity (h)	723		0.00	1.00	158		0.00	0.58	240		0.00	0.75	325		0.00	1.00	*** ^a,b^
Social interactions (h)	723		-0.83	2.00	158		-0.29	2.63	240		-0.50	2.00	325		-1.00	3.00	* ^a,b^
News checking (h)	723		0.75	1.17	158		0.50	1.00	240		0.51	1.00	325		1.00	1.50	NS
Mid-sleep (MSFsc)^‡^	723		0.19	0.71	158		0.17	0.72	240		0.92	0.50	325		0.26	0.86	** ^a,b^
Social jetlag (h)^‡^	723		-0.08	0.50	158		-0.17	0.5	240		0.00	0.41	325		-0.13	0.50	NS
Sleep latency (min.) ^‡^	723		0.00	10.71	158		0.00	10.71	240		0.00	1.96	325		8.57	30.00	*** ^a,b,c^
Night sleep efficiency (%)^‡^	723		-2.10	8.02	158		0.42	6.16	240		-0.98	5.02	325		-5.29	10.90	*** ^a,b,c^
Sleep duration (h/24) ^‡^	723		0.00	1.14	158		0.53	-10.10	240		0.00	0.64	325		-0.14	1.46	*** ^a,b,c^
**During lockdown assessments:**																	
Poor sleep: PSQI (%); global PSQI (0-21) ^§^	723	54.5%	6.00	5.00	158	30.4%	4.00	3.00	240	35.0%	5.00	3.00	325	80.6%	8.00	5.00	*** ^d^
Using sleep medications past month)^§^		12.3%			158	8.9%			240	7.1%			325	17.8%			*** ^d^
High dream recall frequency ^‖^		51.3%			158	53.8%			240	39.6%			325	58.8%			*** ^d^
High dream intensity ^‖^		25.3%			150	16.3%			220	12.7%			305	38.4%			*** ^d^
Anxiety (%); absolute HADS_D (0-21) ^¶^		39.8%	6.00	6.00		20.5%	6.00	5.00	237	26.3%	6.00	5.00		60.7%	7.00	6.00	*** ^d^
Depression (%); absolute HADS_A (0-21) ^¶^		29.7%	5.00	5.00		14.7%	5.00	5.00	237	19.8%	5.00	5.00		45.6%	5.00	6.00	*** ^d^
Social/emotional loneliness score (0-12) ^#^	706		2.00	3.00	155		2.00	6.00	236		2.00	3.00	315		3.00	3.00	*** ^a,b^
Lonely some-almost all of the past week ^††^	695	50.2%			154	39.0%			232	40.9%			311	62.7%			*** ^d^
Poorer mood (lockdown vs. pre-lockdown)	703	50.6%			145	22.8%			236	36.9%			297	65.5			*** ^d^

***p < .0001, **p < .001, *p < .05, NS, non-significant

Items from:

^†^ SF-36

^‡^ µMunich Chronotype Questionnaire

^§^ Pittsburgh Sleep Quality Index

^‖^ The Mannheim dream questionnaire

^¶^ Hospital Anxiety and Depression Scale (HADS)

^#^ Gierveld’s loneliness scale

^††^ General Social Survey

Post hoc analyses indicating areas of significance: Kruskal–Wallis: a = worse sleep vs. better sleep; b = worse sleep vs. no change; c = better sleep vs. no change; Chi Square: d = worse sleep vs. no change or better sleep, e = no change vs. better sleep or worse sleep.

Other variables considered at the univariate level but were skewed or non-significant and therefore not further included such as: ethnicity, marital status, length of time in lockdown or isolation, number of coresidents, qualifications, home type, access to garden, carer status, pet ownership, weekly consumption of tobacco, alcohol, or caffeine.

### Changes to sleep, mood, and behaviour during lockdown compared to pre-lockdown

**Figure 1.**
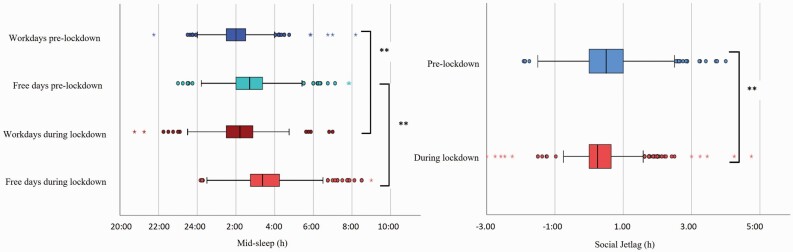
(a) Box plots of mid-sleep timing (hour) pre-lockdown (dark blue and cyan) and during lockdown (dark red and red) indicating significant (*p* < .001) differences for both work- and free-days (*N* = 723). (b) Box plots of social jetlag (hours) for pre-lockdown (blue) and during lockdown (red, *N* = 723, *p* < .001). A positive social jetlag value indicates later sleep timing on free days than on workdays.

Sleep timing differed significantly from pre-lockdown estimates to current lockdown estimates with bedtimes occurring 15 min later on workdays and 30 min later on free days (*p <* .001). Median get-up times also moved 43 min later on workdays and 30 min later on free days (*p* <.001) compared to pre-lockdown estimates. Despite this, weighted 24-hour sleep duration (see Methods) remained a stable 8 h (*p* = .161), but most likely because participants spent more time in bed, night-time sleep latency increased, and sleep efficiency decreased (a median change of 4.2%, *p* < .001, see Table 2). Changes of mid-sleep on work- and free days prior and during lockdown are also illustrated in Figure 1a. The mid-sleep time on free days (MSFsc) occurred 15 min later during lockdown and social jetlag decreased by 15 min (see Figure 1b; *p* < .001), reflecting that the differences between mid-sleep on free and on workdays became smaller. Mood was reportedly worse in 50.6% of the participants compared to pre-lockdown (Table 1). Furthermore, while time spent physically active remained stable, participants reported spending significantly less time outside in daylight (median 1 h less per day), less time socially interacting (median 1 h less per day), and more time keeping up with the news (1 h more per day) during lockdown compared to pre-lockdown estimates (Table 2; *p* < .001).

**Table 2.** Sleep timing on work- and free days and weighted sleep summary data (µMunich Chronotype Questionnaire; MSFsc = corrected for sleep durations on free days), and daytime activities compared pre- and during lockdown using Wilcoxon Signed ranks (***p < .0001, **p < .001, *p < .05, NS, non-significant)

	Pre-lockdown			During lockdown			Wilcoxon ranks	
	*N*	Median	IQR	*N*	Median	IQR	*z*	*P*
Workdays (*n*)	723	5.00	1.00	723	5.00	3.00	-7.96	***
Free days (*n*)	723	2.00	1.00	723	2.00	3.00	7.96	***
**On workdays:**								
Bedtime (h)	650	22.00	1.25	583	22.25	1.50	3.96	***
Sleep onset (h)	650	23.00	1.25	583	23.00	1.50	7.50	***
Wake time (h)	650	6.50	1.00	583	7.00	1.50	10.71	***
Sleep duration (h)	650	7.50	1.00	584	7.75	1.50	3.75	***
Get-up time (h)	650	6.79	1.08	583	7.50	1.25	15.56	***
Time in bed (h)	650	8.50	1.00	583	9.00	1.52	13.05	***
Mid-sleep (h)	650	2.00	1.00	583	2.21	1.30	6.40	***
**On free days:**								
Bedtime (h)	723	22.50	1.00	723	23.00	1.50	3.43	**
Sleep onset (h)	723	23.08	1.50	723	23.50	1.67	8.81	***
Wake time (h)	723	7.25	1.50	723	7.50	2.00	3.39	**
Sleep duration (h)	723	8.00	1.25	723	8.00	1.75	-9.25	***
Get-up time (h)	723	8.00	1.75	723	8.50	2.00	9.64	***
Time in bed (h)	723	9.50	1.25	723	9.50	1.75	5.24	***
Mid-sleep (h)	723	2.43	1.38	723	3.38	1.50	19.32	***
**Sleep summary**								
Mid-sleep (MSFsc)	723	3.00	1.16	723	3.25	1.41	9.86	***
Social jetlag (h)	723	0.50	1.00	723	0.25	0.66	−10.04	***
Nap time (h)	280	0.67	1.00	282	0.75	1.00	1.95	NS
Sleep duration 24 h (h)	723	8.00	1.26	723	8.08	1.68	1.40	NS
Sleep efficiency (%)	723	88.92	10.17	723	86.09	13.65	−11.50	***
**Waking activities**								
Daylight exposure (h)	723	2.00	2.00	715	1.00	1.25	−5.02	***
Physical activity (h)	723	1.00	1.50	715	1.00	1.50	−0.37	NS
Social interactions (h)	722	3.00	3.00	714	2.00	3.00	−10.12	***
News checking (h)	722	1.00	1.50	715	2.00	2.00	16.93	***

Boxes in Figures 1(a) and (b) represent the data range from quartiles 1–3; the vertical line representing the median value; the whiskers and lines representing cut offs (i.e. minimum and maximum values, excluding outliers); and circles and asterisks representing mild and extreme outliers respectively).

### Assessed items relating to the lockdown phase only

Survey items relating to the lockdown phase only included the PSQI score, which indicated that 54.5% of the sample were experiencing “poor sleep” (as determined by a score >5), 12.3% reported using some form of sleep medication or aid within the last month. The HADS indicated that 39.8% scored within range for anxiety and 29.7% depression. High dream recall frequency (i.e. several times a week – almost every morning) was reported by 51% of participants and 25.7% of the participants reported that their dream content was quite – very intense during lockdown (Table 1).

### Associations of self-rated sleep changes with sleep timing, dreaming, waking activities, mood, anxiety, and loneliness

**Figure. 2.**
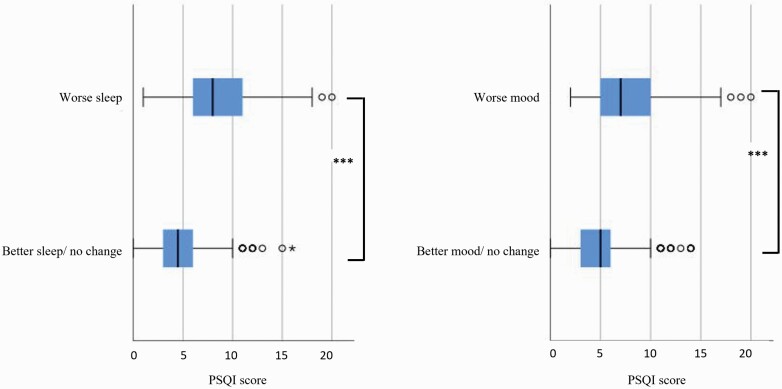
(a). Box plots of PSQI scores for those reporting worse sleep (upper graph) during lockdown (median = 8.0, IQR = 3.0) vs. better sleep or no change (lower graph; median = 4.5, IQR = 3), *n* = 723, Kruskal–Wallis = 200.78, *p* <.0001. (b). Box plots of PSQI scores for those reporting worse mood (upper graph) during lockdown (median = 7.5, IQR = 6.0) vs. better sleep or no change (lower graph; median = 5.0, IQR = 3), *n* = 723, Kruskal–Wallis = 119.32, *p* <.0001.

Almost half (45.0%) of participants rated their sleep quality as worse since the beginning of the COVID-19 pandemic restrictions, and 21.9% rated their sleep as better, while 33.2% reported no change. The distribution percentages and statistics of key demographic variables, sleep timing, dreaming, waking activities, mood, anxiety, and loneliness status are shown in Table 1, grouped by perceived change in sleep quality (3 options). This shows that those who rated their sleep as worse during lockdown were significantly more likely to have poorer self-reported mood and health, have greater changes to the timing of sleep and waking activities, with shorter daylight exposure, and less time spent in physical activities, and social interactions (*p* < .001). Those reporting worsening sleep during lockdown were also more likely to report more frequent and vivid dreams as well as feeling of loneliness. Global PSQI scores were significantly higher (indicating poorer sleep quality) among those who self-rated their sleep or mood as worsening with lockdown (Figure 2, a and b respectively, *p* < .001). Table 3 shows a binomial regression model concerning independent predictors for reporting worse sleep quality during lockdown (as opposed to having no change or better sleep).

**Table 3.** Binomial generalised linear models identifying factors associated with reporting worse sleep. *N* = 558, regression coefficients (B), and Odds Ratios (OR) are shown with upper and lower Confidence Intervals (95% CI) and *p* values (*p* values < 0.05 are shown in bold)

		Worse sleep (reference: no change or better sleep)			
Variable	Description	B	OR	95% CI	*P*
Age	20–85 years	0.00	1.00	0.98–1.01	0.66
Female*	82.3%	−0.17	0.84	0.46–1.57	0.59
Works shifts or variable hours*	16.7%	−1.14	0.32	0.16–0.62	**<0.001**
Health status^†^	1 (excellent) – 5 (poor)	0.02	1.02	0.76–1.37	0.89
Mental illness*	33.2%	−0.01	0.99	0.59–1.65	0.97
**Change in activities and sleep during lockdown vs. pre-lockdown:**					
Daylight exposure (L10 ratio change)^**^	−17.0 – 19.0 h	−0.39	0.68	0.36–1.24	0.22
Physical activity (L10 ratio change)^**^	−19.0 – 19.0 h	0.00	1.00	0.63–1.62	1.00
Social activity change	−14.0–14.0 h	0.07	1.07	0.99–1.16	0.09
Mid-sleep change^‡^	−3.8 – 6.4 h	0.19	1.20	0.89–1.63	0.22
Sleep latency change^‡^	−84.3 – 115.0 min	0.03	1.03	1.01–1.05	**<0.001**
Sleep efficiency change^‡^	−64.5 – 46.7 %	−0.05	0.95	0.91–0.98	**<0.01**
24hr sleep change^‡^	−10.1 – 7.26 h	0.10	1.11	0.90–1.41	0.38
***During lockdown:***					
Sleep disturbance score^§^	0 (none) – 21 (severe)	0.19	1.21	1.08–1.35	**<0.01**
Uses sleep aids currently^§^	12.3% (ref: no sleep meds)	−0.34	0.71	0.32–1.58	0.40
Dream recall^#^	0 (never) – 6 (almost every morning)	0.06	1.06	0.88–1.28	0.53
Dream intensity^#^	0 (not at all) – 4 (very intense)	0.46	1.59	1.20–2.11	**<0.01**
Anxiety score^¶^	0 (no) – 21 (severe)	0.09	1.10	1.02–1.18	**0.01**
Depression score^¶^	0 (no) – 21 (severe)	0.07	1.07	0.98–1.18	0.12
Loneliness score^ç^	1 (no) – 6 (severe)	−0.08	0.92	0.78–1.09	0.35
Mood poorer (during lockdown vs. pre-lockdown)	50.6%	0.69	1.99	1.20–3.32	**0.01**

*For results from categorical variables: Female (reference: male/other); works shifts/variable daytime hours (reference: daytime without shifts); existing mental illness (reference: no mental illness); mood poorer during lockdown (reference: no change or better mood during lockdown)

Items derived from:

^†^ SF-36

^‡^ µMunich Chronotype Questionnaire (log-transformed values)

^§^ The Pittsburgh Sleep Quality Index

^#^ The Mannheim dream questionnaire

^¶^ The Hospital Anxiety and Depression Scale

^ç^ the Gierveld loneliness scale

^**^Change in times associated with daylight and physical activity were analysed on log-transformed (L10) of the ratio of change due to skewed data

Boxes in Figures 2a and 2b represent the data range from quartiles 1–3; the vertical line representing the median value; the whiskers and lines representing cut offs (i.e. minimum and maximum values, excluding outliers); and circles and asterisks representing mild and extreme outliers respectively). Higher PSQI scores indicate greater sleep disturbance, with scores > 5 defined as “poor sleepers”.

When controlling for factors which were significant at the univariate level (see Table 1), those reporting worse sleep had increased sleep latency [from a median of 18.8 min (IQR 1.89) to 30.0 min (IQR = 45)] compared to those reporting no change or better sleep [who’s median sleep latency remained at 15 min (IQR = 20; *p* < .001, Table 3]. Those participants reporting worse sleep also had reduced sleep efficiency [from 89.0 (IQR = 9.7) down to 81.8% (IQR = 1)] compared to those with no change/better sleep, whose median sleep efficiency remained between 88% and 89% (IQR = 11; *p* = .004). Scores of sleep disturbance (PSQI) remained independently associated with reports of worse sleep during lockdown (OR = 1.2, *p* = .001) and increments of dream intensity were significantly associated reporting worse sleep (OR: 1.6; *p* = .001).

In addition, those participants reporting worse sleep during lockdown were 32% *less* likely to be among those working shifts or variable work patterns during lockdown compared to those who worked in the daytime without shifts (*p* = .001). Higher scores related to anxiety were associated with worsening sleep (OR 1.1; *p* = .015). Participants with worse sleep were 2.0 times more likely to report mood deterioration since lockdown (*p* = .01). Note, younger age, poorer self-rated health, having a mental health condition, and a reduction in daylight exposure were also associated with reporting worsening sleep. However, these did not remain significant once variables associated with sleep status and mood were controlled for (see Supplementary Table A: model 2 vs. models 3 and 4).

## Discussion

This study examined subjective sleep quality, timing, and duration as well as relationships with waking activities, mood, anxiety, loneliness during the initial 2020 lockdown and compared it to the pre-lockdown state. Results are novel as pertain to those locked-down in NZ, a country where disease outbreak was dramatically stunted compared to elsewhere [15, 16]. Participants spent more time in bed and had later sleep phase during lockdown compared to pre-lockdown estimates. They also had a reduction in social jetlag between work and free days, but reported worse mood, less time spent in daylight and with social interactions, and longer times checking news.

These findings are comparable to research concerning lockdowns in other countries [3, 4] and are likely related to increased flexibility of waking and social schedules, as well as sleeping within personal preferences particularly on “workdays”. For example Blume et al [6] noted a 13 min decrease in social jet lag; and Rezaei and Grandner [5] reported reductions in bedtime variability between work- and free days, particularly in younger participants (derived from FitBit data). Despite more time spent in bed, total sleep duration across the 24-hour day did not significantly change in the present sample. Rather, participants reported increased sleep latency and poorer sleep efficiency. These changes to sleep timing are indicative of insomnia-like sleep disturbances and maybe fragmented sleep that has been reported elsewhere with the onset of the pandemic and its associated stressors such as fear of disease, additional family care requirements, and financial worries [6, 11, 13, 24].

Almost half (45%) of the participants rated their sleep as worse during, when compared to pre-lockdown. Those rating their sleep as worse were also significantly more likely to have increases in sleep latency and declines in sleep efficiency since the commencement of lockdown. Correspondingly, they also had higher PSQI scores during lockdown indicating a higher prevalence of sleep disturbances compared to those rating their sleep as unchanged or improved, corroborating other data on COVID-19 related restrictions [3, 4].

Those working shifts or variable work patterns were significantly *less* likely to report worse sleep during lockdown compared to those with a stable work pattern. Previous research identifies shift workers as more susceptible to poor sleep and sleep practices [25]. But despite this, those who are already managing unstable sleep patterns (i.e. shift workers), may also be better prepared to manage the changes to schedules (i.e. those enforced during lockdown) and therefore less likely to report a negative effect on their sleep at this time [26]. 

Environmental light exposure, physical activity, and social engagement act as “zeitgebers” (time cues) on circadian sleep–wake regulation as well as informing mood status and wellbeing [27, 28]. In the present sample, time spent exposed to light and social interactions decreased and news checking significantly increased during lockdown, while physical activity remained stable. Such changes have been identified as moderating sleep quality during the pandemic [6, 24]. While reductions in light, physical activity, and social engagements were associated with reports of worsening sleep in the present sample, their direct effect on worse sleep disappeared after controlling for mood and demographic variables. This may be due to the mediating impact that age, and physical and mental health status can also have on sleep together with waking activities and work.

Regarding mood, those reporting worse sleep during lockdown had higher scores indicative of anxiety and were twice as likely to rate their mood as worse during lockdown compared to pre-lockdown. Depression and feelings of loneliness were also more prevalent among those with worse sleep compared to better or unchanged sleep during lockdown. Anxiety and deteriorations of mood during lockdown remained independently related to worse sleep even after adjusting for other factors. The relationship between sleep and mood is well defined and considered bidirectional [12]. Factors such as social routines and connections play important mediating roles in the ecology of sleep and mental health [1]. Negative impacts of the pandemic situation on mood and mental wellbeing have been identified elsewhere [3, 11, 29]. These findings provide an example of how the impact of lockdown with very low disease outbreak in NZ had both positive or negative effects on sleep and how mood, particularly anxiety, influenced this and vice versa, since we cannot extract unidirectional causality from our data. The nuanced aspects of such relationships may be informed by the qualitative aspects of the open-ended comments which will be reported elsewhere.

Those rating their sleep as worsening had correspondingly more intense and vivid dreams. Specific dream content was not collected in the present study. However, studies have found dream content to be more bizarre or unpleasant during the pandemic and more likely to contain themes of disease, apocalypse, and personal protective equipment since the onset of the pandemic [13, 30, 31]. Such changes in dream experience are also commonly reported during times of personal transition, stress, or trauma [14]. Changes to dream intensity may also be associated with spending likely more time in bed during lockdown [32, 33] as well as increased emotional and behavioural possessing required to process and adapt to the rapidly changing situation [34].

The COVID-19 lockdown dealt an acute period of social change and stress where, for many, sleep was compromised. Such changes were related to psychological impact of the situation, changed schedules, and environment, as well as mood. The present findings are unique because, unlike research from other countries, the impact of strict social restrictions was assessed without the high infection rates seen elsewhere, therefore controlling for a key aspect considered to jeopardise sleep and wellbeing. Within this NZ sample, the impacts of lockdown on sleep, while present, were not as pronounced as found in data from countries with greater disease outbreak [11]. For example, Varma and colleagues’ study of 1745 respondents (predominantly from Australia, India, United Kingdom, South Africa, United States) found that 57% (12% more than this sample), reported poorer sleep since the pandemic and, using higher cut off score (>8) within the PSQI, 47% were defined as poor sleepers. Within the current NZ sample, only 31.6% of respondents scored within this more severe range. Varma and colleagues [9] also noted a mean sleep duration of just 6.59 h (SD 1.46) during lockdown, 1.42 h less than the present NZ sample (8.01 h, SD 1.43). With sleep durations averaging around a healthy 8-hours both pre-and during lockdown, this may explain why substantial increases in time spent asleep were not found here compared to other studies using self-report [6] or objectively recorded data [5]. Similarly, no detriments in sleep duration were found among those rating their sleep as worsening during lockdown (while Varma et al. did [9]).

The present findings may also reflect a less pronounced impact of the pandemic and lockdown experience on anxiety and mood within this NZ sample compared to elsewhere. Even though the prevalence of being classified as “anxious” or “depressed” was raised in our survey responses when compared to normative HADS data among other non-clinical prepandemic samples (e.g. Crawford et al. recorded 33.2% within the mild-severe range for anxiety and 11.4% for depression [35]), it was still lower than assessed during the elsewhere during the pandemic. For example, Martinez et al [36] found that 48.3% of their Brazilian participants were classified as having depression symptoms and 82.6% as having anxiety symptoms. Using different scales, Varma et al [9] and Morin et al [11] also reported greater prevalence of mood disorders. These discrepancies may reflect less negative affect of the COVID-19 situation among New Zealanders during this initial lockdown given the reduced threat of COVID-19 contagion compared to elsewhere. Perception of disease risk was not measured here so could not be controlled for. But, given the distinctive trajectory of the disease in NZ compared to elsewhere, this is an area of future consideration. Morin et al [11] found that the number of COVID-related deaths by country was independently related to indicators of insomnia, indicating important other social, economic, and cultural interconnections with regards to sleep, mood, and disease status.

This research offers a unique insight into the impact of a COVID-19 lockdown within NZ. However, some considerations are warranted. Being an online survey collected within a limited timeframe, our sample does not derive from a representative population sample. The majority were female, of NZ European ethnicity, and highly educated. Previous research concerning sleep in NZ has identified disparities by socioeconomic status and ethnicity, with Māori and Pacific Islanders being more likely to experience sleep disruptions [37]. Socioeconomic disparities compromised living and sleeping environments, poor physical and mental health as well as reduced access to healthcare are all thought to contribute to poor sleep [37, 38]. Such populations have also been identified as marginalised with regards to the COVID-19 pandemic [15, 39, 40]. Therefore, the prevalence of sleep and mood disturbances within the NZ population is likely greater than reported here.

The study’s methods also limit the generalisability of the findings. For instance, the cross-sectional nature restricts measures of prospective changes in sleep and wellbeing. This was somewhat addressed by including pre-lockdown estimates for key items such as sleep status. The incorporation of objective assessments (e.g. using actigraphy) and sleep, dream, and mood diaries may have enhanced this however were outside of the scope of the current study.

To conclude, the present study presents data on changes to sleep status and related factors within the unique situation of NZ, where a strict lockdown was implemented in 2020 while disease outbreak remained low. Despite some of the assumed burden of the pandemic being reduced in this situation, rates of problematic sleep and mood were still raised. These findings point to the unique roles of social routines, norms, and connections with regards to sleep as well as the interconnected relationship to mental health. All factors which require support within the early stages of crises.

## Supplementary Material

zpac017_suppl_Supplementary_Material

## Notes

Institute where the study was performed: Sleep/Wake Research Centre, Massey University, Wellington, New Zealand.
