# Supplementary material for: Sleeping in a bubble: factors affecting sleep during New Zealand’s COVID-19 lockdown
Source: Sleep Adv. 2022 May 16;3(1):zpac017. doi: 10.1093/sleepadvances/zpac017 (PMC9383987; doi:10.1093/sleepadvances/zpac017)
Supplement: zpac017_suppl_Supplementary_Material [file zpac017_suppl_supplementary_material.pdf]

Supplementary Material:

Sleeping in a Bubble: Factors Affecting Sleep During New Zealand's COVID-19 Lockdown

Authors and affiliations:
Rosemary Gibson PhD [1,2], Harshi Shetty[2], Mikaela Carter MSc [2], & Mirjam Münch PhD [2,3, 4]

[1] School of Psychology, Massey University, Palmerston North, New Zealand
[2] Sleep/Wake Research Centre, Massey University, Wellington, New Zealand*
[3] Research Centre for Hauora and Health, Massey University, Wellington, New Zealand
[4] Centre for Chronobiology, University Psychiatric Clinics, Basel, Transfaculty Research Platform Molecular and Cognitive Neurosciences, Switzerland

*Institute where the study was performed

Correspondence:
R. Gibson, School of Psychology, Massey University Private Bag 11 222 r.gibson@massey.ac.nz

Table A. Binomial generalised linear models identifying factors associated with reporting worse sleep during lockdown (ref: no change/better sleep)

	Model 1				Model 2				Model 3				Model 4			
**Variable**	**B**	**OR**	**95% CI**	**P**	**B**	**OR**	**95% CI**	**P**	**B**	**OR**	**95% CI**	**P**	**B**	**OR**	**95% CI**	**P**
Age	-0.02	0.98	0.97-0.99	**0.01**	-0.02	0.98	0.97-1.00	**0.03**	-0.01	0.99	0.97-1.01	0.17	0.00	1.00	0.98-1.01	0.66
Female^	0.24	1.27	0.77-2.09	0.35	0.26	1.30	0.79-2.16	0.30	-0.04	0.96	0.53-1.75	0.89	-0.17	0.84	0.46-1.57	0.59
Works shifts/variable^	-0.82	0.44	0.26-0.73	**<0.0001**	-0.89	0.41	0.24-0.68	**<0.0001**	-1.15	0.32	0.16-0.60	**<0.0001**	-1.14	0.32	0.16-0.62	**<0.0001**
Health status[1]	0.38	1.46	1.19-1.81	**<0.0001**	0.34	1.40	1.13-1.74	**<0.0001**	0.06	1.06	0.80-1.40	0.70	0.02	1.02	0.76-1.37	0.89
Mental illness^	0.59	1.80	1.23-2.65	**<0.0001**	0.59	1.81	1.23-2.68	**<0.0001**	0.16	1.17	0.72-1.89	0.53	-0.01	0.99	0.59-1.65	0.97
**Change in activities and sleep during lockdown (vs. pre-lockdown):**																
Daylight exposure change Δ					-0.55	0.58	0.35-0.94	**0.03**	-0.49	0.61	0.34-1.08	0.09	-0.39	0.68	0.36-1.24	0.22
Physical activity change Δ					-0.17	0.85	0.59-1.21	0.36	-0.07	0.94	0.61-1.45	0.77	0.00	1.00	0.63-1.62	1.00
Social activity change					0.00	1.00	0.94-1.06	0.92	0.06	1.06	0.99-1.15	0.11	0.07	1.07	0.99-1.16	0.09
Mid sleep change[2]									0.20	1.22	0.91-1.64	0.19	0.19	1.20	0.89-1.63	0.22
Sleep latency change[2]									0.03	1.03	1.01-1.04	**<0.0001**	0.03	1.03	1.01-1.04	**<0.0001**
Sleep efficiency change[2]									-0.04	0.96	0.93-0.99	**0.02**	-0.05	0.95	0.91-0.98	**<0.0001**
24-hr sleep change[2]									0.04	1.05	0.86-1.31	0.68	0.10	1.11	0.90-1.41	0.38
**During lockdown:**									0.27	1.31	1.19-1.46	**<0.0001**				
Sleep disturbance score[3]									-0.37	0.69	0.32-1.45	0.32	0.19	1.21	1.08-1.35	**<0.0001**
Uses sleep aids currently[3]									0.02	1.02	0.86-1.23	0.79	-0.34	0.71	0.32-1.58	0.40
Dream recall[4]									0.55	1.73	1.32-2.29	**<0.0001**	0.06	1.06	0.88-1.28	0.53
Dream intensity[4]													0.46	1.59	1.20-2.11	**<0.0001**
Anxiety score[5]													0.09	1.10	1.02-1.18	**0.01**
Depression score[5]													0.07	1.08	0.98-1.18	0.12
Loneliness score[6]													-0.08	0.92	0.78-1.09	0.35
Mood poorer^													0.69	1.99	1.20-3.32	**0.01**

The table shows significant p values are shown in bold, regression coefficients (B), odds ratios (OR), confidence intervals (CI) for 4 different regression models

^ References for categorical variables: female (ref: male/other); works shifts/variable daytime hours (ref: daytime without shifts); existing mental illness (ref: no mental illness); mood poorer during lockdown (ref: no change or better mood).

Items derived from: [1] SF-36; [2]µMunich Chronotype Questionnaire, corrected for sleep duration on free days (MSFsc; log-transformed values); [3] The Pittsburgh Sleep Quality Index; [4]The Mannheim dream questionnaire; [5].The Hospital Anxiety and Depression Scale, and [6] , the Gierveld's loneliness scale.

Δ Change in times associated with daylight and physical activity were imputed as the log-transformed (L10) of the ratio of change due to enhance model specificity
